# Identification of Plasma Thrombopoietin Level and Its Significance in Patients with Aplastic Anemia and Myelodysplastic Syndrome

**DOI:** 10.1055/s-0043-1771456

**Published:** 2023-07-28

**Authors:** Mengying Zhang, Gaochao Zhang, Fangfang Xu, Mengyuan Liu, Xifeng Dong, Weiwei Qi, Huaquan Wang

**Affiliations:** 1 Department of Hematology, Tianjin Medical University General Hospital, Tianjin, People's Republic of China; 2 Department of Hematology, Beijing Friendship Hospital, Capital Medical University, Beijing, People's Republic of China

**Keywords:** aplastic anemia, myelodysplastic syndrome, thrombopoietin

## Abstract

**Objective** Our objective was to investigate the concentration of plasma thrombopoietin (TPO) in patients with aplastic anemia (AA) and myelodysplastic syndrome (MDS), as well as its relationship with patients' responses to recombined human TPO (rhTPO) therapy.

**Methods** We detected the concentration of plasma TPO in 31 patients with AA, 27 patients with MDS, and 11 normal controls using enzyme-linked immunosorbent assay.

**Results** The median concentration of plasma TPO in patients with AA, MDS, and controls was (841.08 ± 768.64), (212.41 ± 338.93), and (35.09 ± 18.21) pg/mL, respectively. The TPO concentration in patients with AA and MDS was significantly higher than that in controls ( *p* < 0.05). The median platelet (PLT) counts were (184 ± 34) ×10 ^9^ /L in the control group and (24 ± 19) ×10 ^9^ /L and (80 ± 71) ×10 ^9^ /L in AA and MDS patients, respectively. Negative correlations were found between plasma TPO concentration and PLT counts as well as megakaryocytes in bone marrow ( *p* < 0.05). In AA patients treated with rhTPO, a negative correlation was observed between increased PLT counts and pretreatment TPO levels ( *p* < 0.05).

**Conclusion** Plasma TPO concentration in AA and MDS was significantly higher than that in normal controls. Plasma TPO was negatively correlated with peripheral blood PLT counts and bone marrow megakaryocyte counts. The pretreatment TPO level may serve as a prognostic indicator for the therapeutic effect of rhTPO in AA patients.

## Introduction

Aplastic anemia (AA) is a benign bone marrow failure syndrome that often causes pancytopenia, leading to symptoms such as anemia, bleeding, and infection. 1 Myelodysplastic syndrome (MDS) is a malignant clonal disease that often causes cytopenia. Anemia, bleeding, and infection are also common symptoms that can be life-threatening for patients. 2 While the mechanisms of thrombocytopenia caused by these two diseases are not the same, both present difficulties in clinical treatment. Therefore, it is of great significance to study the mechanisms of thrombocytopenia in depth and provide targeted treatments.

Thrombopoietin (TPO) is a cytokine produced by the liver and kidney that is mainly involved in the growth and development of megakaryocytes and the regulation of platelet (PLT) production. 3 TPO activates the downstream signaling pathway through its receptor c-MPL, which not only promotes the proliferation and differentiation of megakaryocytes but also those of hematopoietic stem/progenitor cells. 4 5

In this study, we detected plasma TPO concentration in patients with AA and MDS as well as normal controls and explored differences in plasma TPO concentration between bone marrow failure disease patients and normal controls. We also analyzed the correlation between TPO concentration and PLT counts, evaluated the efficacy of recombined human TPO (rhTPO) in treating AA patients, and analyzed the correlation between pretreatment plasma TPO level and the efficacy of rhTPO.

## Subjects and Methods

### Subjects

A total of 69 patients were enrolled in this study, including 31 AA patients (17 males and 14 females; age range, 8–67 years; median age, 39 years), 27 MDS patients (17 males and 10 females; age range, 28–75 years; median age, 54 years), and 11 healthy controls (6 males and 5 females; age range, 14–56 years; median age, 29 years). Among the AA group, 14 patients were treated with antithymocyte globulin and cyclosporine-based immunosuppressive therapy combined with rhTPO. According to the International Prognostic Scoring System, the MDS group was divided into low-risk (score 0; *n* = 2), intermediate-1 (score 0.5–1.0; *n* = 11), intermediate-2 (score 1.5–2.0; *n* = 5), and high-risk (score ≥ 2.5; *n* = 9) subgroups. This study was approved by the Ethics Committee of our hospital, and all subjects provided informed consent.

Plasma TPO detection: ethylenediaminetetraacetic acid-anticoagulant blood was centrifuged at 1,500 g/min for 10 minutes, and then, the plasma was aspirated and cryopreserved at −80°C for next use. Plasma TPO concentrations were measured using an enzyme-linked immunosorbent assay (ELISA) with a MultiSciences TPO ELISA kit (EK-11522) according to the manufacturer's instructions. PLT counts were measured using a blood cell analyzer. All patients included in this study were newly diagnosed and had not yet received any treatment prior to plasma TPO detection.

### Statistical Processing

Relevant statistical analyses were performed using SPSS 22.0. All data were expressed as mean ± standard deviation. The independent-sample *t* -test was used for the significance of mean differences, and the Spearman correlation test for correlation analysis. The relevant data were analyzed by the chi-square test. The cut-off values were determined using the receiver operating characteristic (ROC) curve and verified with the chi-square test. *p* < 0.05 was considered statistically significant.

## Results

### Plasma Thrombopoietin Concentration Increased in Aplastic Anemia and Myelodysplastic Syndrome Patients

**Fig. 1:**
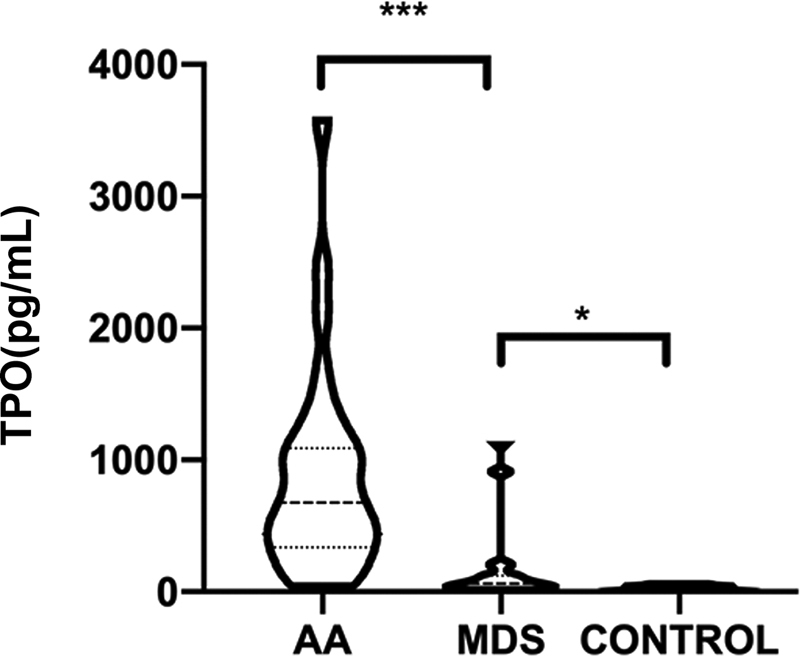
The plasma TPO concentration of patients with aplastic anemia, myelodysplastic syndromes, and controls. * *p* < 0.05, *** *p* < 0.01.

The plasma TPO concentration was 35.09 ± 18.21 pg/mL in the normal control group, 841.08 ± 768.64 pg/mL in the AA group, and 212.41 ± 338.93 pg/mL in the MDS group. The concentration of TPO in the AA group was significantly higher than that in normal control and MDS groups, respectively ( *p* < 0.001, *p* = 0.012). Compared with normal control group, the TPO level in the MDS group was increased, with a statistically significant difference ( *p* < 0.05; Fig. 1 ).

### Correlation between Thrombopoietin levels and Platelet Counts

**Fig. 2:**
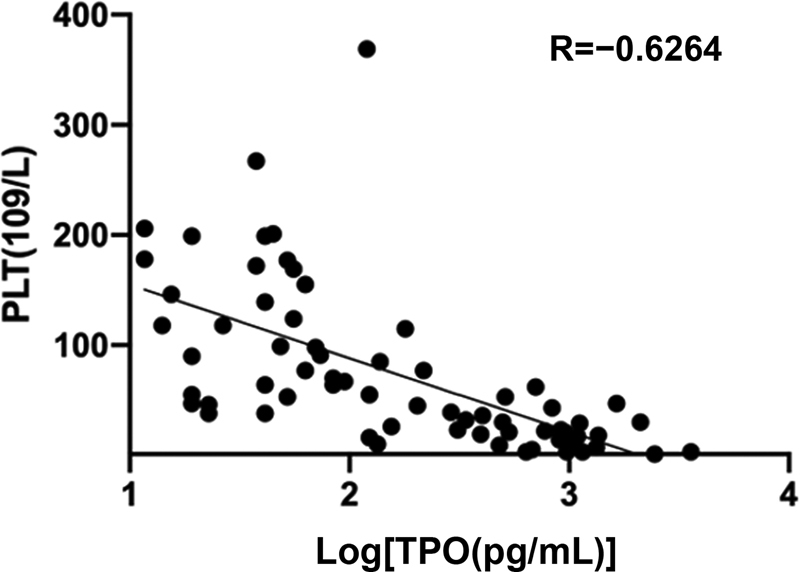
Correlation between plasma TPO concentration and PLT, Spearman correlation test, *p* < 0.05.

**Fig. 3:**
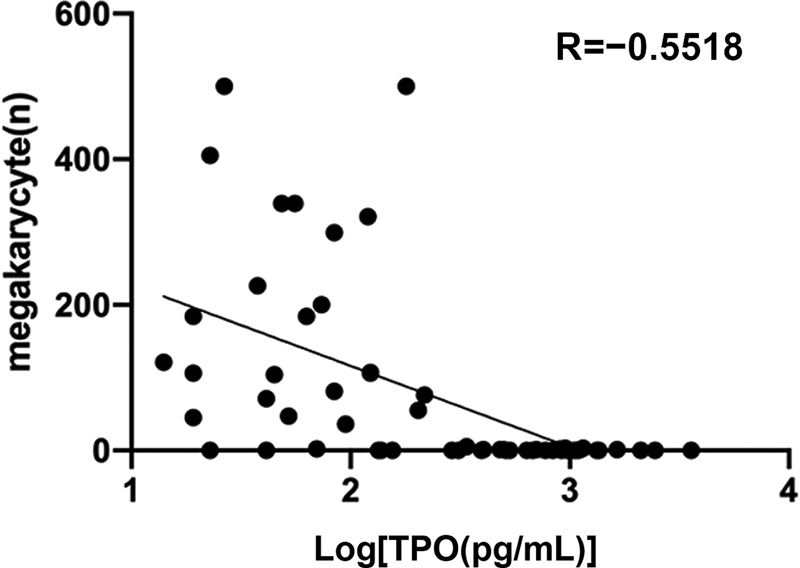
Correlation between plasma TPO concentration and megakaryocytes.

The mean PLT count was 184 ± 34 × 10 ^9^ /L in the normal control group, 28 ± 25 × 10 ^9^ /L in the AA group, and 89 ± 71 × 10 ^9^ /L in the MDS group. The correlation analysis showed a significantly negative correlation between plasma TPO concentration and PLT counts ( Fig. 2 ). Furthermore, comparing the patients with similar PLT counts in the AA group and MDS group showed that the plasma TPO concentration was lower in patients with more megakaryocytes ( Fig. 3 ).

### Efficacy of Recombined Human Thrombopoietin

**Fig. 4:**
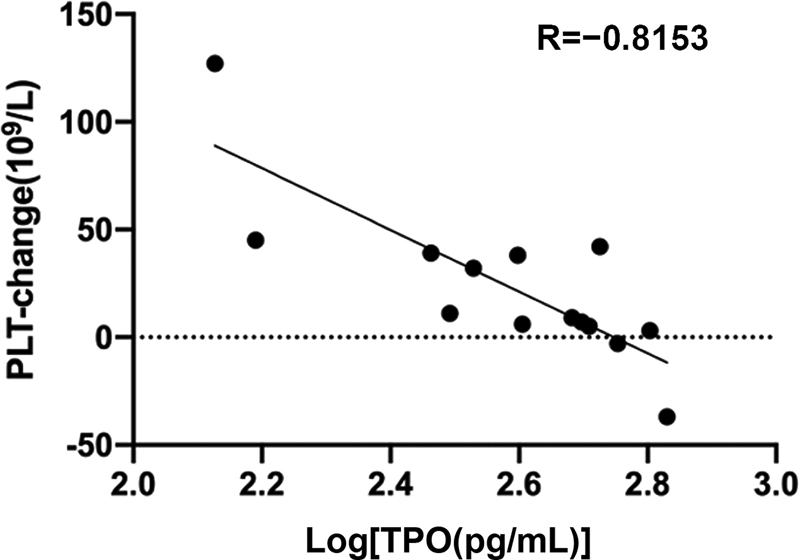
Plasma TPO level before treatment and PLT changes after immunosuppressive therapy in AA patients (8 weeks later).

In AA patients treated with rhTPO, the increase of PLT count 8 weeks after treatment was found to have a negative correlation with pretreatment TPO concentration ( *p* < 0.05; Fig. 4 ). We also determined a cut-off value of 396.38 pg/mL for TPO concentration using an ROC curve analysis. Among the 14 patients, 8 (57.14%) had TPO concentration greater than 396.38 pg/mL and only 1 (12.5%) of them did not require PLT transfusion 8 weeks after treatment. On the contrary, six (52.85%) patients had TPO concentration lower than 396.38 pg/mL, and five (83.33%) of them were able to discontinue PLT transfusion 8 weeks after treatment ( *p* = 0.044).

## Discussion

TPO is a glycoprotein hormone that plays a crucial role in regulating PLT production. It acts by binding to and activating its receptor, c-MPL, which is expressed on the surface of hematopoietic stem cells, megakaryocytes, and PLTs. Through this mechanism, TPO can stimulate the proliferation, differentiation, and maturation of these cells, ultimately leading to an increase in PLT production. 4 Under the stimulation of TPO, hematopoietic stem cells differentiate into megakaryocytes by regulating and expressing different transcription factors (GATA1, FOG1, RUNX1, FLI1, and NF-E2). Most of the TPO in plasma binds to c-MPL on the surface of PLTs and a small part binds to megakaryocytes. When PLT count decreases, the level of free TPO in plasma increases, stimulating hematopoietic progenitor cells in the bone marrow to differentiate into megakaryocyte lines to produce more PLTs. The reverse is also true, that is, the formation of negative feedback regulation of PLT production. 6 Mice defective in the c-mpl or the TPO gene show a greater than 80% reduction in their megakaryocyte and PLT numbers, without affecting the other hematopoietic lineages. 7 8 Interestingly, the regulation of TPO levels appears to be mainly mediated by c-MPL, rather than transcriptional control. In patients with congenital amegakaryocytic thrombocytopenia, a condition characterized by a lack of megakaryocytes and PLTs due to c-MPL deficiency, the level of plasma TPO is significantly increased. 9 This suggests that c-MPL plays a crucial role in the negative feedback regulation of TPO levels and that its absence can lead to dysregulation of TPO production.

Our results showed that plasma TPO levels in AA and MDS patients were significantly higher than those in the normal control group, and they were negatively correlated with PLT counts, which was consistent with the previous results. 9 10 11 Studies have revealed that the c-MPL expression on cells of AA patients decreased significantly compared with the normal control group, and the significantly increased plasma TPO levels in AA patients compared with the normal group may be related to the significant reduction of CD34 cells and megakaryocytes in the bone marrow and PLTs in the peripheral blood, thus reducing the damage to TPO and causing an increase in TPO level in AA patients. 12 13 Decker et al 14 have founded that TPO can down-regulate the expression of its receptor c-mpl mRNA in a dose-dependent manner. The higher the additional rhTPO, the lower the expression of c-mpl mRNA, reflecting the diversity of cytokine regulation. That is, TPO is not regulated at the transcriptional level but can regulate its own function by down-regulating the receptor c-mpl mRNA. On the contrary, the number of PLTs and megakaryocytes can regulate the gene expression of TPO through a feedback mechanism, so as to maintain the dynamic balance of megakaryocyte development and PLT generation in the body. 15 The plasma TPO level in MDS patients was slightly higher than that in that normal control group and was still lower than that in the AA group after PLT count correction. Seiki et al 16 have reported that the expression levels of TPO increased in MDS patients and showed differences in different groups, confirming that increased plasma TPO levels were associated with a favorable prognosis of bone marrow failure.

Fontenay-Roupie et al 17 have demonstrated that the expression of c-MPL increased in mononuclear cells of MDS patients. The results of our study showed that the PLT count of the AA group was significantly lower than that of the MDS group, and the number of bone marrow megakaryocytes in the MDS group was higher than that in the AA group, resulting in more damage to TPO compared with AA patients. Therefore, the TPO level in the MDS group was lower than that in the AA group. By comparing the patients with similar the PLT count in the AA group and the MDS group, we found that the more the megakaryocytes, the lower the plasma TPO concentration, which can also support this conclusion. That is, the bone marrow megakaryocytes of AA patients had poor regeneration, and the TPO binding to c-MPL was reduced, so the plasma TPO level was significantly increased. Moreover, other study showed that the expression of TPO mRNA is roughly the same in different diseases. 18 Consequently, we believe that the differences in TPO concentration between AA, MDS, and normal controls are mainly caused by different bone marrow megakaryocytes, CD34 cells, and peripheral blood PLTs but are not related to the pathogenesis of diseases.

We and other scholars have found that TPO combined with immune suppressive therapy can significantly reduce the need for blood product infusion and have a significant effect on hematopoiesis in AA patients, with fewer adverse reactions, which can lower the cost of hospitalization. 19 20 We analyzed AA patients receiving TPO treatment and determined their TPO cut-off value by analyzing the ROC curve, revealing that patients below the cut-off value had better efficacy, and the level of plasma TPO before treatment could predict the efficacy, which may have guiding significance for the clinical treatment and efficacy prediction of AA patients. Although the plasma TPO level of AA patients was higher than that of the normal controls, we believe that the normal hematopoietic stem cells and bone marrow megakaryocytes of AA patients reduce significantly compared with those of the normal control group, and the increased TPO level is not enough to mobilize hematopoietic stem cells and megakaryocytes to produce PLTs. Therefore, we speculate that high-dose exogenous TPO will play a role in the hematopoietic recovery of AA patients. The sample size in our study is relatively small, which may limit the generalizability of the results. Therefore, we considered that further studies should be conducted to investigate the efficacy and safety of rhTPO treatment in a larger cohort of AA patients.

In conclusion, the level of plasma TPO in patients with AA and MDS increases, which is correlated with PLT and bone marrow megakaryocyte. In addition, the level of plasma TPO before treatment can predict the efficacy of rhTPO for PLT elevation in AA.
